# Therapeutic Drug Monitoring for Aminoglycosides: Not Yet Readily Available in Japanese University Hospitals

**DOI:** 10.31662/jmaj.2021-0134

**Published:** 2021-12-15

**Authors:** Hideharu Hagiya, Fumio Otsuka

**Affiliations:** 1 Department of General Medicine, Okayama University Graduate School of Medicine, Dentistry and Pharmaceutical Sciences, Okayama, Japan

**Keywords:** aminoglycoside, antimicrobial resistance, gentamicin, tobramycin, amikacin, therapeutic drug monitoring

As leading hospitals, national and public university hospitals have a mission to treat a large number of patients with intractable and/or critical diseases. Aminoglycosides broadly cover Gram-negative glucose non-fermenters, such as *Pseudomonas aeruginosa* and *Acinetobacter* species, by inhibiting the function of the ribosome 30S subunit ^(1)^. Thus, these agents are regarded as key drugs in the treatment of patients with sepsis, especially in cases of nosocomial onset and in those who have been isolated with antimicrobial-resistant pathogens ^(2), (3)^.

To ensure the safety and effectiveness of aminoglycoside therapy, it is essential to maintain appropriate serum concentrations through therapeutic drug monitoring (TDM) ^(4)^. To avoid renal and cochlear impairments, the trough level needs to be kept reasonably low. To obtain clinical effectiveness, a high peak level should be achieved. Aminoglycosides are water-soluble drugs that generally penetrate the urinary tract, ascites, pleural fluid, and articular fluid. However, pharmacokinetically, they do not diffuse well into organ tissues, especially the lower respiratory tract, prostate, and biliary systems, and spread poorly within the central nervous system. Thus, a fine adjustment of serum concentrations is essential when administering aminoglycosides for infections of these organs. Aminoglycoside-resistant organisms may emerge as a consequence of overexpression of the excretion pump and decreased binding to ribosomes by enzymatic modification ^(1)^. Thus, maintaining appropriate serum concentrations can also prevent the emergence of antimicrobial resistance (AMR). The purpose of this study was to understand the current availability of the in-house ability to measure aminoglycosides in national and public university hospitals in Japan.

In March 2021, we distributed a questionnaire to 43 national and public university hospitals in Japan, asking about the status of TDM for aminoglycoside agents (gentamicin [GM], tobramycin [TOB], and amikacin [AMK]). For comparison, we also collected the measurement status of the following four other antimicrobial drugs: vancomycin, teicoplanin, arbekacin, and voriconazole. Ethics committee approval for the publication of the data was obtained from the Institutional Review Board of Okayama University Hospital (No. 2106-004). The need for informed consent was waived because detailed data on the individual institutions were anonymized.

A total of 30 institutions responded to the survey, of which data from 27 institutions (62.8%), excluding one specialized dental hospital and two hospitals that have withdrawn their participation in the data analysis, were included in the analysis (Table 1). Serum concentrations of aminoglycosides were measured in the clinical laboratory, pharmacy, or in both at 10 (37.0%), 11 (40.7%), and 6 (22.2%) institutions, respectively. The number (proportion) of institutions with in-hospital assays of aminoglycoside agents were 15 (55.6%), 5 (18.5%), and 3 (11.1%) for GM, TOB, and AMK, respectively. The number (proportion) of institutions that outsourced serum aminoglycoside testing was 12 (44.4%), 13 (48.1%), and 23 (85.2%) for GM, TOB, and AMK, respectively. Several institutions did not offer even outsourced testing: 0 for GM, 9 (33.3%) for TOB, and 1 (3.7%) for AMK, respectively. Of the 27 institutions, the number (percentage) of aminoglycoside agents tested in-hospital was 12 (44.4%), 9 (33.3%), 4 (14.8%), and 2 (7.4%) for no agent, one agent, two agents, and three agents, respectively.

**Table 1.** Monitoring of Aminoglycosides and Other Antimicrobial Agents at 27 National and Public University Hospitals in Japan.

	In-hospital		Outsourced		Not available	
	n	(%)	n	(%)	n	(%)
Aminoglycosides						
Gentamicin	15	55.6	12	44.4	0	0.0
Tobramycin	5	18.5	13	48.1	9	33.3
Amikacin	3	11.1	23	85.2	1	3.7
Other antimicrobials						
Vancomycin	27	100.0	0	0.0	0	0.0
Teicoplanin	22	81.5	5	18.5	0	0.0
Arbekacin	9	33.3	15	55.6	3	11.1
Voriconazole	8	29.6	19	70.4	0	0.0

By comparison, the availability of in-hospital monitoring of agents used to treat methicillin-resistant *Staphylococcus aureus* agents was 100% for vancomycin, 81.5% for teicoplanin, and 33.3% for arbekacin. Serum measurements of voriconazole were outsourced in about three-fourths of the institutions.

Our data revealed that TDM of aminoglycosides is still not readily available at many national and public university hospitals in Japan, although clinical guidelines recommend a fine adjustment of the drugs according to the TDM results ^(5), (6)^. The optimization of clinical indications, dosages, and target concentrations of aminoglycosides has yet to be fully established ^(7)^. However, aminoglycosides are the drugs of choice in frequently encountered clinical situations, such as urinary tract infections, due to AMR organisms in outpatient settings, and as both an empirical and definitive therapy for patients in a state of shock.

In this age of AMR, promoting antimicrobial stewardship and avoiding under-dosing of antimicrobials by means of TDM is pivotal to prevent the emergence of AMR pathogens. With the growing population of difficult-to-treat cases, practicing TDM-guided individualized treatment is required. Attainment of the pharmacokinetic/pharmacodynamic target is of great value in delivering appropriate drug doses, achieving a high treatment success rate, and improving the prognosis of patients without unnecessary adverse effects. To apply it fully in clinical practice, TDM should be conducted at appropriate intervals, without delay ^(4)^. Close collaboration between clinicians, microbiologists, and pharmacists is essential for the development of therapeutic strategies to manage fluctuating drug levels by adjusting the dose of antimicrobial therapy. Therefore, we believe that an in-house measurement system should be established in tertiary medical institutions such as national and public university hospitals.

## Article Information

### Conflicts of Interest

None

### Acknowledgement

We would like to thank Editage (www.editage.jp) for assisting with editing this manuscript.

### Author Contributions

All authors meet the ICMJE authorship criteria. HH was responsible for the study design, data analyses, and writing of the manuscript. FO supervised the study. All authors approved the final report.

### Approval by Institutional Review Board (IRB)

The ethics committee approval for the publication of the data was obtained from the Institutional Review Board of Okayama University Hospital (No. 2106-004). The need for informed consent was waived because detailed data on the individual institutions were anonymized.
